# Proximity of immune and tumor cells underlies response to BRAF/MEK-targeted therapies in metastatic melanoma patients

**DOI:** 10.1038/s41698-021-00249-1

**Published:** 2022-01-20

**Authors:** Chi Yan, Sheau-Chiann Chen, Gregory D. Ayers, Caroline A. Nebhan, Joseph T. Roland, Vivian L. Weiss, Douglas B. Johnson, Ann Richmond

**Affiliations:** 1 Tennessee Valley Healthcare System, Department of Veterans Affairs, Nashville, TN USA; 2 Vanderbilt University School of Medicine, Department of Pharmacology, Nashville, TN USA; 3 Department of Biostatistics, Vanderbilt University Medical Center, Nashville, TN USA; 4 Division of Hematology and Oncology, Department of Medicine, Vanderbilt University Medical Center, Nashville, TN USA; 5 Departments of Surgery and Pediatrics and the Epithelial Biology Center, Vanderbilt University School of Medicine, Nashville, TN USA; 6 Department of Pathology, Microbiology, and Immunology, Vanderbilt University Medical Center, Nashville, TN USA; 7 Vanderbilt Ingram Cancer Center, Vanderbilt University Medical Center, Nashville, TN USA

**Keywords:** Cancer therapeutic resistance, Melanoma

## Abstract

Acquired resistance to BRAF/MEK-targeted therapy occurs in the majority of melanoma patients that harbor BRAF mutated tumors, leading to relapse or progression and the underlying mechanism is unclear in many cases. Using multiplex immunohistochemistry and spatial imaging analysis of paired tumor sections obtained from 11 melanoma patients prior to BRAF/MEK-targeted therapy and when the disease progressed on therapy, we observed a significant increase of tumor cellularity in the progressed tumors and the close association of SOX10^+^ melanoma cells with CD8^+^ T cells negatively correlated with patient’s progression-free survival (PFS). In the TCGA-melanoma dataset (*n* = 445), tumor cellularity exhibited additive prognostic value in the immune score signature to predict overall survival in patients with early-stage melanoma. Moreover, tumor cellularity prognoses OS independent of immune score in patients with late-stage melanoma.

Approximately 50% of melanoma tumors harbor activating mutations in the BRAF oncogene, rendering these tumors susceptible to treatment with BRAF/MEK inhibitors^1^. While BRAF/MEK-targeted therapy induces rapid response and improves survival, the majority of patients eventually experience disease progression^2^. Although several predictive and prognostic biomarkers for clinical responses have been identified, such as overall mutation burden, pathway-specific mutations (e.g., BRAF/MEK/CDKN2A), and absolute lymphocyte/neutrophil count (ALC/ANC) ratio, additional biomarkers are needed to better understand the process of progression in patients receiving BRAF inhibitor with or without MEK inhibitor for melanoma therapy^3,4^.

We previously identified 11 patients treated with BRAF and/or MEK inhibitors at the Vanderbilt-Ingram Cancer Center with matched tumor sections obtained pre-treatment and at disease progression^5^. Of these patients, the median (range) age was 47 (21–77) and five were men (45.5%). Of our cohort, four (36.4%) were treated with BRAF inhibitors alone, four (36.4%) were treated with dual BRAF and MEK inhibitors, and three (27.3%) were treated with single‐agent BRAF inhibitors followed by dual therapy (BRAF/MEK or BRAF/PI3K). All patients had a complete or partial response, with the exception of one patient (9%) with a mixed response, and the median PFS was 11.6 months for the entire cohort (Supplementary Table 1).

Next, multiplex immunohistochemistry (MxIHC) and whole-tumor imaging spatial analyses were performed on these tumor sections to determine the composition and location of immune cells (e.g., CD8^+^ T cells and CD11c^+^ dendritic cells [DC]) in close proximity to SOX10^+^ melanoma cells in parallel with an assessment of expression of markers for tumor immune surveillance mechanisms (e.g., costimulatory molecules CD40 and CD80 for immune cell activation^5,6^), or association with clinical parameters pre- and post-treatment (Fig. 1). We observed a significant increase in SOX10^+^ melanoma cells in the progressed tumors compared to the tumors prior to BRAF/MEK-inhibition from the same patient (Fig. 2a). While the frequency of pre-existing SOX10^+^ melanoma cells prior to treatment was not associated with PFS, a greater increase in SOX10^+^ melanoma cells from baseline to progression was associated with shorter PFS under BRAF/MEK inhibition treatment (Spearman *r* = −0.842, *p* = 0.004) (Fig. 2b). We speculated that melanoma cells may suppress immune cell responses for immune escape and disease progression. However, there was no alteration in the number of CD8^+^ T cells or CD11c^+^ DCs in the progressed tumors compared to the tumors prior to therapy (Supplementary Fig. 1). Neither the pre-existing frequency nor change of frequency of CD8^+^ T cells or CD11c^+^ DCs was associated with PFS (Supplementary Fig. 1). Intercellular sensing and communication (e.g., soluble cytokines/chemokines) requires close proximity (~40 μm) of interacting cells^7^. We next counted the SOX10^+^ melanoma cells that paired with CD8^+^ T cells and normalized the pairs to the total CD8^+^ T-cell number since CD8^+^ T-cell number was not altered due to therapy. The data show that those patients with a high number of SOX10^+^ melanoma cells at progression and a higher number of SOX10 & CD8^+^T-cell pairs had a significantly shorter PFS (Spearman *r* = −0.75, *p* = 0.025). This effect was most pronounced for patients where melanoma cells were paired within 45 μm of CD8^+^ T cells (Spearman *r* = −0.783, *p* = 0.017) (Fig. 2c). No significant differences were observed in CD40 and CD80 content. There is an extensive data overlap between patients with and without prior immunotherapy or immunotherapy-chemotherapy combination before the BRAF/MEK-therapy, suggesting our observation is likely independent of prior chemo-/immuno- therapy status (Supplementary Fig. 2). Given the uneven distribution of SOX10^+^ cells in the tumor sections, visual estimates of percentage tumor nuclei in the region-of-interests (ROIs) were determined by pathologists who were blinded to treatment (Supplementary Fig. 3). These ROIs (1–3 per tumor) which exhibited viable tumor cells and reliable SOX10 expression were selected by the pathologists. Consistent with whole-slide SOX10 quantitation, the change of %SOX10^+^ cells in ROIs confirmed a negative correlation with patient’s PFS (Spearman *r* = −0.733, *p* = 0.0311). We also observed a moderate positive correlation between the visually estimated percentage tumor nuclei (generally >60%) and the computer-assisted SOX10^+^ cell density quantitation (generally 10–60%). Nevertheless, there was no alteration of visually estimated percentage tumor nuclei in the progressed tumors compared to the tumors prior to BRAF/MEK-inhibition. As such, percentage tumor nuclei does not correlate to the patient’s PFS. Visual estimation alone is known to be variable and inaccurate^8^. Our whole-slide MxIHC-based SOX10% results determined by computer-assisted cell density quantitation are in line with the observations of AstroPath multispectral imaging platform and may highlight the benefit of computer-assisted cell density quantitation to capture treatment-induced responses and antibody cocktail staining of melanoma cell markers to reduce false-negative signals^9^.

**Fig. 1:**
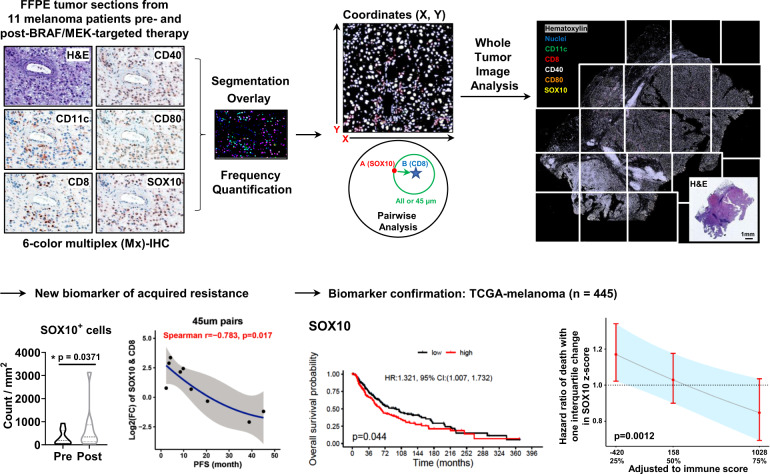
Overall workflow of MxIHC and spatial analysis. The analysis started with brightfield MxIHC staining and whole-tumor image scanning. For each patient slide, image segmentation was performed to quantify the frequency of targeted cells and to obtain coordinates. The coordinate list from each slide was fed to pairwise analysis to calculate the relative intervals between targeted cells. The association between the survival and immune score signature or new biomarkers (SOX10 and MLANA), as well as adjusted HR plot based on the added value of a new marker into the immune score signature, were assessed in Skin Cutaneous Melanoma (TCGA, Firehose Legacy) dataset.

**Fig. 2:**
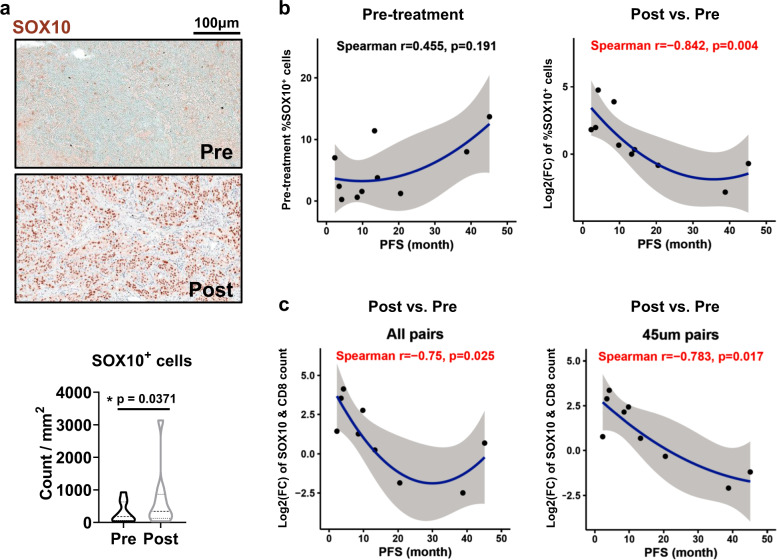
Close association of SOX10^+^ melanoma cells with CD8^+^ cells negatively correlated with the PFS of patients treated with BRAF/MEK-targeted therapy. Paired advanced melanoma samples from 11 patients (pre- and post-treatment of BRAF-targeted therapy) were analyzed. **a** Representative images and the violin plot of (SOX10^+^) melanoma cell count per mm^2^ in tumors with Wilcoxon signed-rank test. **b**, **c** Scatter plots for the association between variables were shown with Spearman’s rank correlation test, a quadratic regression line WITH its 95% CI. **b** Frequency (%) was normalized to the count of all nucleated cells in each tumor. **c** The number of paired SOX10^+^ melanoma cells within a distance of 45 μm of a CD8^+^ cell, or in reference to any distance, was normalized to the number of CD8^+^ cells in each tumor. The log2-converted fold-change (FC) values were used to measure changes in progressed tumors compared to tumors prior to therapy.

Immune score based on the ESTIMATE method was previously generated from 11 different tumor types^10^ and confirmed in melanoma^11^ to infer tumor purity, stromal and immune cell admixture from expression data in The Cancer Genome Atlas (TCGA). We further showed that OS was associated with immune score stratified by disease stage in melanoma (*n* = 445) (Fig. 3a). In contrast, increased SOX10 z-scores were associated with decreased OS of patients with late-stage (*n* = 192; hazard ratio [HR] = 1.186; 95% confidence interval [CI] = 1.006–1.397; p = 0.03), but not early-stage melanoma (*n* = 217; HR = 1.203; 95%CI = 1.008–1.436; *p* = 0.192) (Fig. 3b). Notably, compared to the main effect of immune score only, the multivariable analysis presented an additive interaction of SOX10 with an immune score to predict OS in patients with early-stage (likelihood ratio [LR] test, *p* = 0.005), but not late-stage melanoma (LR test, *p* = 0.554) (Fig. 3c, d). Together, our data revealed significant interaction and an additive effect of the prognostic value of SOX10, confirmed by another melanoma marker MLANA (Supplementary Fig. 4), with an immune score in early- but not late-stage melanoma. Thus, for early-stage patients with a low immune score, the OS of melanoma patients with tumors of high SOX10 (Wald test, *p* = 0.0012) or MLANA (Wald test, *p* = 0.0051) was worse than that of those with low levels of SOX10 or MLANA. For late-stage patients, increased SOX10 (log-rank test, *p* = 0.03) or MLANA (log-rank test, *p* = 0.018) prognoses worse OS independent of immune score.

**Fig. 3:**
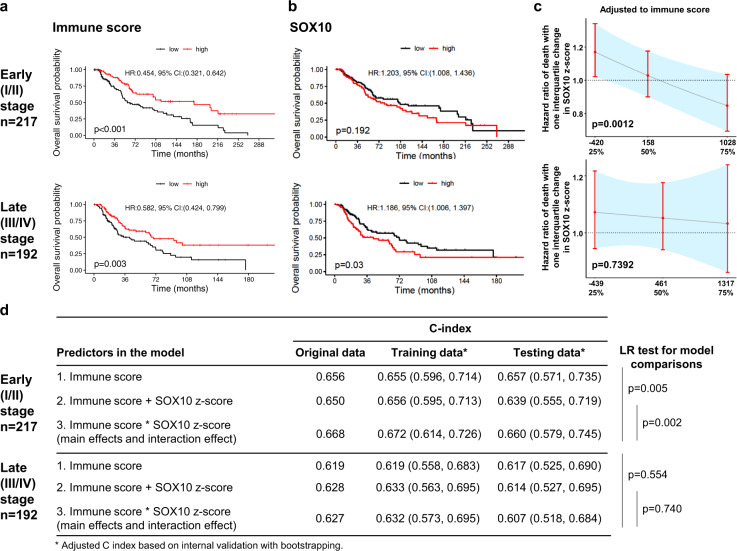
Prognostic value and interaction of SOX10 and immune score to predict OS in patients with melanoma. **a**, **b** Survival (Kaplan–Meier) plots by predictors. The mortality risk (HR of death) was calculated based on per interquartile change in continuous predictors. **c** HR of death per interquartile change in SOX10 z-score adjusted to immune score. **d** Likelihood ratio (LR) test showed an improved prognostic performance of immune score via adding SOX10 to predict OS in patients with stage I/II melanoma.

In the TCGA-melanoma cohort, SOX10^hi^CD8^low^ tumors were associated with a significantly worse OS compared to SOX10^low^CD8^hi^ tumors in melanoma patients with either early (I/II, *p* < 0.001) or late (III/IV, *p* = 0.0036) tumor stage (Supplementary Fig. 5). As expected, we found that CD8^hi^ inflamed tumors exhibited higher levels of *PD1*, *PDL1*, *IFNG*, and *GZMB*, compared to the CD8^low^ tumors (*p* < 0.001) (Supplementary Fig. 6). In addition, SOX10^hi^CD8^hi^ tumors exhibited reduced levels of *IFNG* (*p* < 0.001) and *GZMB* (*p* = 0.01) compared to SOX10^low^CD8^hi^ tumors. Similarly, SOX10^hi^CD8^low^ tumors exhibited a significant reduction of *IFNG* (*p* = 0.008) and a trend of reduction in *GZMB* (*p* = 0.39), compared to SOX10^low^CD8^low^ tumors. Together, these data suggest that SOX10^hi^/CD8^low^ is associated with a poor anti-tumor immune response and poor OS in melanoma.

Several mechanisms have been reported to suppress effector T-cell responses via co-inhibitory molecule interaction with melanoma cells, including PD-L1:PD-1^12^, CTLA-4:B7-1/2^13^, LSECtin:LAG-3^14^, CD155:TIGIT^15^, etc. Using the YUMM3.3 (BRAF^mut^) melanoma model in C57BL/6 mice, we found that ~30% of CD45^−^ cells, including melanoma cells, are PD-L1^+^, while ~80% of CD8^+^ T cells are PD-1^+^ in the tumor microenvironment (TME) (Supplementary Fig. 7a). Two weeks of anti-PD-1 treatment resulted in a significant (>50%) inhibition of tumor growth (Supplementary Fig. 7b). Anti-PD-1 treatment does not alter the frequency of total CD45^+^ leukocytes, total CD3^+^ T cells, or CD8^+^ T cells in the TME (Supplementary Fig. 7c). However, while ~10% of CD8^+^ T cells are activated (CD69^+^) in the tumors treated with isotype control IgG, anti-PD-1 treatment increased the frequency of activated CD8^+^ T cells to ~40% in the TME (Supplementary Fig. 7c). These data confirmed the detrimental role of the PD-L1:PD-1 axis between melanoma cell:Tc-cell in melanoma.

In this retrospective study, we performed MxIHC and subsequent whole-tumor imaging spatial analyses of tumors obtained from 11 melanoma patients prior to BRAF/MEK-targeted therapy and after the disease progressed. The data revealed that at the time of progression, the close association of SOX10^+^ melanoma cells with CD8^+^ T cells negatively correlated with PFS of melanoma patients and may indicate a potential mechanism for acquired resistance to BRAF/MEK-targeted therapy. The observed increase of tumor cellularity in progressed melanoma tumors could be either a result or a cause of the escape from BRAF/MEK-inhibition. Additional on-treatment biopsies, as well as assessment of the genetic profile of the tumor cells that expanded post-treatment, would be essential to further explore whether there was an expansion of a treatment-resistant clone that led to poor PFS or was it the density of the tumor cells in relation to the immune effector cells per se that was associated with poorer PFS. Since SOX10 was associated with OS of late-stage melanoma patients in the TCGA dataset, it is plausible that the SOX10 expression level is a prognostic marker in melanoma, and is not linked to any specific treatment response. Using a preclinical mouse model, we showed the detrimental role of PD-L1:PD-1 axis between melanoma cell:Tc-cell in melanoma. Besides cell–cell interaction mechanisms, tumor cells may secrete immunosuppressive cytokines/chemokines and/or compete with immune cells in the microenvironment for the components required for their own metabolism, further inhibiting immune cell functions^16^. Indeed, CD8^+^ T cells in primary melanoma were spatially distant from proliferating (Ki67^+^) tumor cells compared to nondividing (Ki67^−^) tumor cells, suggesting that rapidly growing primary tumors may suppress and/or exclude CD8^+^ T cells or fail to produce factors that recruit these cells into the tumor^17^.

We acknowledge that there are several limitations to our study. First, this study was conducted at a single center with a small sample size, which may result in institution‐specific biases. Second, the clinical data were assessed retrospectively and not in a controlled, prospective fashion. Finally, the melanoma patients received BRAF inhibitor with or without MEK inhibitor. However, we observed a consensus association between outcomes and tumor cellularity irrespective of therapy type. The prognostic value of tumor cellularity was confirmed in the TCGA-melanoma dataset (*n* = 445). Further research is needed to understand the crosstalk between rapidly proliferating melanoma cells and T cells to decipher the biological mechanisms underlying the acquired resistance of BRAF/MEK-targeted therapy.

## Methods

### Patient material

Institutional IRB approval and written informed consent from all patients were obtained before study initiation. All patient donors signed informed consent before providing tissue samples. Patient samples were collected on a tissue-collection protocol approved by the Vanderbilt University IRB. Paired advanced melanoma samples from 11 patients (pre- and post-treatment of BRAF/MEK-targeted therapy) were collected as part of NCT01205815 clinical trial^5^.

### Multiplex immunohistochemistry (MxIHC) assessment and spatial analysis

Immunohistochemical (IHC) staining of 10% buffered formalin-fixed, paraffin-embedded tissue sections was performed by the VUMC Translational Pathology Shared Resource (TPSR). Slides were placed on the Leica Bond Max IHC Stainer. All steps besides dehydration, clearing, and coverslipping were performed on Bond Max. Slides were deparaffinized. Heat-induced antigen retrieval was performed on the Bond Max using their Epitope Retrieval 2 solution for 20 min. Hematoxylin & Eosin (H&E) staining slides were received in PBS. The Shandon Varistain Gemini stainer (A78010402, Thermo, Kalamazoo, MI) was used for H&E visualization. Slides were incubated with primary anti-human antibodies, including anti-CD8 (Cat: MM39-10, McKinney, TX), anti-SOX10 (Cat: PA0813, Cell Marque, Rocklin, CA), anti-CD40 (Cat: ab13545, Abcam, Cambridge, MA), anti-CD80 (Cat: 134120, Abcam, Cambridge, MA), or anti-CD11c (Cat: PA0554, Lecia, Buffalo Grove, IL). The Bond Polymer Refine detection system (DS9800, Leica, Buffalo Grove, IL) and the Vector AEC (3-amino-9-ethylcarbazole) HRP Substrate (SK-2405, Vector Lab, Burlingame, CA) was used for chromogen deposition. Hematoxylin (DS9800, Leica, Buffalo Grove, IL) was used in every cycle of staining for focusing, cell identification, and image registration. After each round of IHC, each slide had a coverslip applied and was imaged at the VUMC Digital Pathology Shared Resource (DHSR). Whole-slide images were captured on an Aperio Versa 200 (Leica). Following image capture, coverslips were carefully removed. The AEC chromogen was bleached by incubation in sequential concentrations of ethanol. The current round of IHC antibodies were removed by heat treatment (95 °C, 15 min). Slides were then returned to TPSR for subsequent rounds of IHC staining. Once all rounds of MxIHC were collected, image processing and image analysis were performed at DHSR. Briefly, whole-slide images were extracted and registered in MatLab (The MathWorks, Inc., Natick, MA). Machine learning of positive AEC signal was performed with Ilastik^18^. Computational single-cell segmentation/identification was performed on the Ilastik probability maps using CellProfiler^19^ and MatLab. Pairwise cell analysis was performed by calculating the Euclidian distance between identified single-cell centroids.

### TCGA-melanoma data and exclusion criteria

Clinical data (*n* = 471) were obtained from cBioPortal (http://www.cbioportal.org/study/ summary?id=skcm_tcga). RNA sequencing-based gene expression profiling in TCGA Skin Cutaneous Melanoma were downloaded from NIH GDC website (https://portal.gdc.cancer.gov/) for survival and the immune score analysis. Patients with overall survival (OS) less than 0, missing overall survival or missing OS status, or patients at stage 0, I/II NOS are excluded. After excluding 26 patients, there were 445 total patients.

### Immune score estimation

Immune scores were derived from RNA-seq data in TCGA Skin Cutaneous Melanoma based on the ESTIMATE algorithm, which were previously generated from 11 different tumor types (not including melanoma) via an R library of estimate package that is available on https://bioinformatics.mdanderson.org/estimate/rpackage.html^10^.

### Mouse tumor models

Animal studies were approved by the Vanderbilt Institutional Care and Animal Use Committee (IACUC) and were performed in accordance with Vanderbilt IACUC guidelines. All animals were housed under pathogen-free conditions at the Vanderbilt Animal Care Facility. C57BL/6 mice were purchased from Jackson Labs. Murine melanoma cell line YUMM3.3 was provided by Marcus Bosenberg (Yale University). The genetics of the YUMM3.3 cell line was verified by RNA-Seq and was free of mycoplasma contamination. Tumor xenografts were established in 7-week-old female mice. For the in vivo melanoma model, mice received 3 × 10^5^ of YUMM3.3 tumor cells in 100 µl of serum-free DMEM medium by subcutaneous injection in the lower back. Mouse body weight was assessed once a week and tumor measurements were taken twice a week with micro-calipers. Tumor volume was estimated as 0.5 x length x width x width. Treatment began when tumors reached ~100 mm^3^ volume on average and continued until tumors in the experiment exceeded 15 mm in diameter or became perforated. Immunotherapy anti-mouse PD-1 (clone: RMP1-14), or equivalent amounts of isotype control Rat IgG2a (clone: 2A3), were administered intraperitoneally at 100 μg per mouse every three days for 2 weeks. All antibodies were purchased from BioXcell (Lebanon, NH).

### Flow cytometric analysis

The details of staining and flow cytometry analysis protocols is according to our previously published methodology^5^. Briefly, cells were incubated with Ghost Dye Violet 510 (Tonbo Biosciences, #13-0870, 1:1,000) to discriminate live/dead cells and washed with PBS containing 1% v/v FBS. After blocking Fc receptors with anti-mouse CD16/CD32 mAb (BD Biosciences, # 553142, 1:50) for 20 min, cells were incubated with target antibodies, including CD45-APC/Cy7 (BioLegend, #103116, 1:250), CD3-PerCP/eFluor 710 (eBioscience, #460032-80, 1:200), CD4-BV421 (BioLegend, #100438, 1:200), CD8-AlexaFlour700 (BioLegend, #100729, 1:500), and CD69-APC (BioLegend, #104513, 1:100). After staining, cells were washed twice in PBS containing 1% v/v FBS and fixed with 1% formalin in PBS. Data were collected using a BD LSR Fortessa flow cytometer and analyzed using FlowJo software (Version 10.5.3). The gating strategy for flow cytometric analysis of tumor samples was shown in Supplementary Fig. 7d.

### Statistical analysis

For BRAF/MEK-targeted therapy clinical data, treatment effects in standard two-group paired experiments were compared using the Wilcoxon matched-pairs signed-rank test. A Spearman’s rank correlation test was used to evaluate the association between PFS and each variable. A scatter plot was shown with a quadratic regression and its 95% confidence interval for visualization.

For univariable analysis of the TCGA Skin Cutaneous Melanoma data, survival curves were estimated using the Kaplan–Meier method and compared between groups including the following variables: gender, stage (I + II vs. III + IV), immune score (low vs. high), SOX10 (low vs. high), MLANA (low vs. high), and CD8 (low vs. high) with the log-rank test. The log-rank test compares the entire survival experience between groups and to see whether the survival curves are identical or not. Please note that median split was used for tuning a continuous variable into a binary variable to visualize the difference between groups. In the survival (Kaplan–Meier) plot, the hazard ratio (HR) of death with 95% CI was reported per interquartile range (IQR) change in continuous predictors based on univariable Cox regression. Taking the survival plot by SOX10 in Fig. 1 as an example, for the unit of one interquartile change of the SOX10 *z*-score, the risk of death increased by 32.1% (HR = 1.321). If the HR is less than 1, for example, 0.454 (like survival plot by the immune score at the early stage in Fig. 3a), the risk of death falls by 54.6% per IRQ change in immune score. When comparing two groups (such as gender), the HR was used to show which groups are more likely to experience an event (death) first. Wilcoxon rank-sum test was used to test the difference in gene expression profiles among four subgroups including SOX10^hi^CD8^hi^, SOX10^hi^CD8^low^, SOX10^low^CD8^hi^, and SOX10^low^CD8^low^. *P* value was adjusted with Bonferroni correction for pairwise comparisons of four subgroups. Redundancy analysis was used to determine how well each variable [immune score, sex, disease stage (I + II vs III + IV), etc.] could be explained by other predicted from the remaining variables. There were no redundant variables detected. Multiple imputation analysis was performed to account for missing data with ten repetitions. It was predetermined that variables with more than 20% missing values were excluded from the analysis (such as BRAF mutations, NRAS mutation, height, and weight). The race was not included in this report because 97% were Caucasian (see in Supplementary Table 2). Variable selection was conducted using regularization methods (elastic-net penalty). (1) Multivariable Cox (proportional hazards) regression was performed for investigating the association between overall survival and predictors and was used to estimate the adjusted hazard ratios. With a continuous predictor, the HR indicates the change in the risk of death per IQR change. Diagnostic tests revealed significant non-proportionality in the disease stage. To ameliorate this violation of modeling assumptions, Cox regression models were stratified by early stage and late stage. (2) The likelihood ratio (LR) test determines the goodness-of-fit between the full model which included interactions between immune score and each marker and the reduced model which contained main effects. A statistically significant interaction implies that the effect of score changes with the level of marker (and the effect of marker differs by the level of score). (3) The concordance index (c index) was used to measure of predictive accuracy and to assess the added value of a new marker (SOX10 and MLANA) where c index is the probability of concordance between the predicted and the observed survival. The c index with bootstrap approach was used to adjust for optimism/overfitting in measures of predictive ability for internal validation. For evaluating the association between OS and an interaction effect of SOX10 and CD8, multivariable cox proportional hazards regression stratified by the disease stage was performed where the SOX10 *z*-score and log2 of CD8 were analyzed to reduce skewness from the predictors. Moreover, multivariable linear regression was used to evaluate the interaction effect of SOX10 z-score and log2 of CD8 on each log2 of gene expressions.

For mouse studies, the progression of tumor volume (mm^3^) over time among groups of mice with or without therapies were compared with a linear mixed-effects regression model to take into account the correlation structure with the repeated measures data within a mouse. A square root or a natural log transformation was implemented to better meet the normality assumptions. The likelihood ratio test was performed to identify statistically significant time by treatment effect. A statistically significant interaction implies that the magnitude of treatment differences depends on the actual day of measurement. Using model-based (least-square) means, the average tumor growth between treatment groups was estimated and compared with the Wald test. R version 4.0.4 was used for statistical analysis.

### Reporting Summary

Further information on research design is available in the Nature Research Reporting Summary linked to this article.

## Supplementary information

Supplementary Figure 1-7 and Supplementary Table 1-2

REPORTING SUMMARY

## Data Availability

The TCGA Skin Cutaneous Melanoma (Firehose Legacy) data that support the findings of this study are available from the website [cBioPortal for Cancer Genomics] (https://www.cbioportal.org/study/summary?id=skcm_tcga). De-identified BRAF/MEK-targeted therapy clinical data are available on request due to privacy restrictions. Data of patient characteristics and response to BRAF-targeted therapy have been presented in aggregate form in Supplementary Table 1. All the data that support the findings of this study are available on request from the corresponding author A.R. (ann.richmond@vanderbilt.edu). The data were not publicly available due to potential compromise of research participant privacy. No data use agreement is required for aggregate data, though access to individual patient-level data requires a data use agreement. No custom code or scripts were used in the generation or analysis of datasets.
