# Supplementary material for: Proximity of immune and tumor cells underlies response to BRAF/MEK-targeted therapies in metastatic melanoma patients
Source: NPJ Precis Oncol. 2022 Jan 20;6:6. doi: 10.1038/s41698-021-00249-1 (PMC8776860; doi:10.1038/s41698-021-00249-1)
Supplement: Supplementary file 1 — Supplementary Figure 1-7 and Supplementary Table 1-2 [file 41698_2021_249_MOESM1_ESM.pdf]

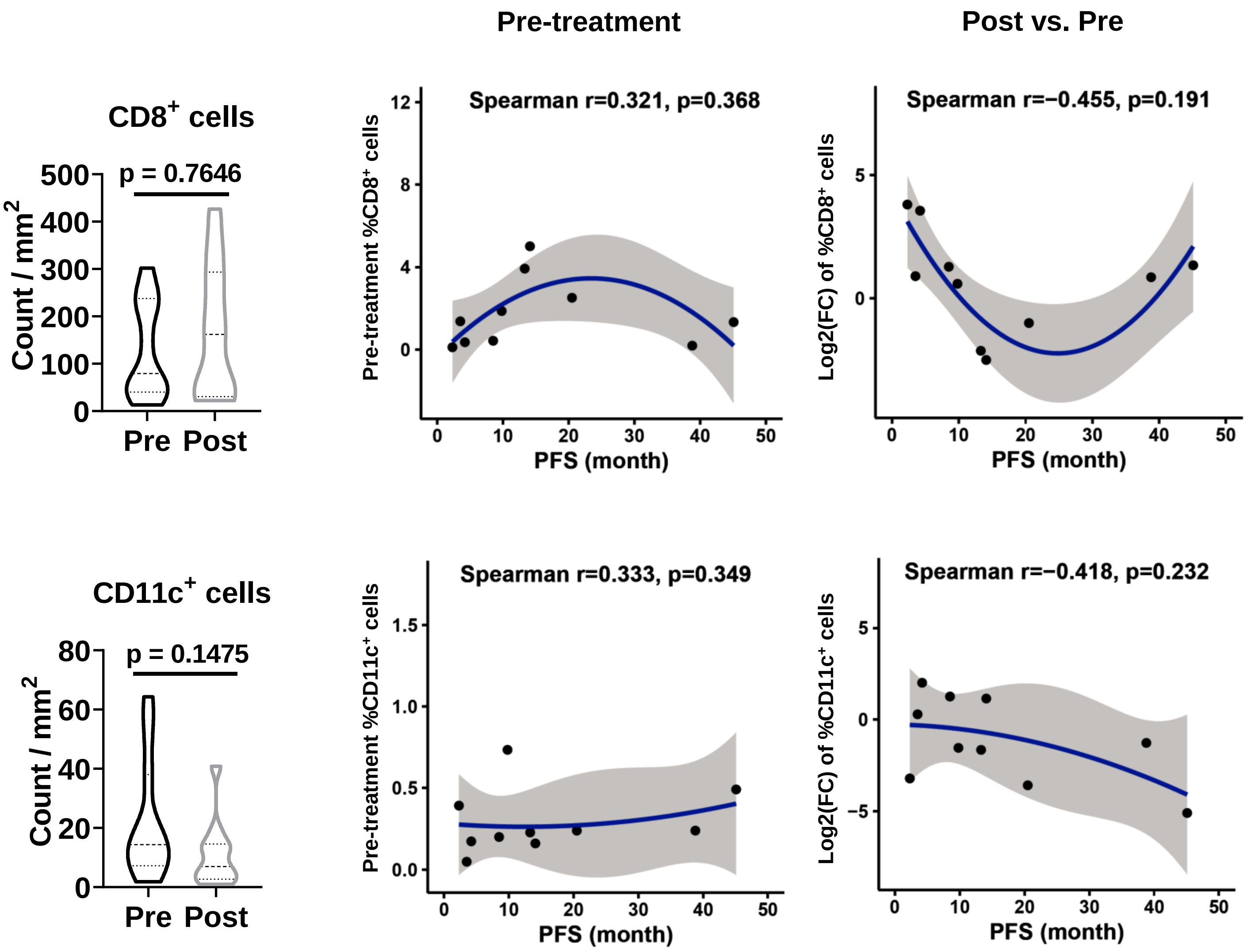

Supplementary Figure 1. Frequency of CD8⁺ and CD11c⁺ cells in melanoma tumors is not correlated with PFS under BRAF/MEK-targeted therapy. Paired advanced melanoma samples from 11 patients (pre- and post-treatment of BRAF-targeted therapy) were analyzed. The violin plots of $CD8^+$ or $CD11c^+$ cell count per mm^2 in tumors with Wilcoxon signed-rank test were shown. Scatter plots with Spearman's rank correlation test were shown with a quadratic regression with 95% confidence interval. Frequency (%) was normalized to the count of all nucleated cells in each tumor.

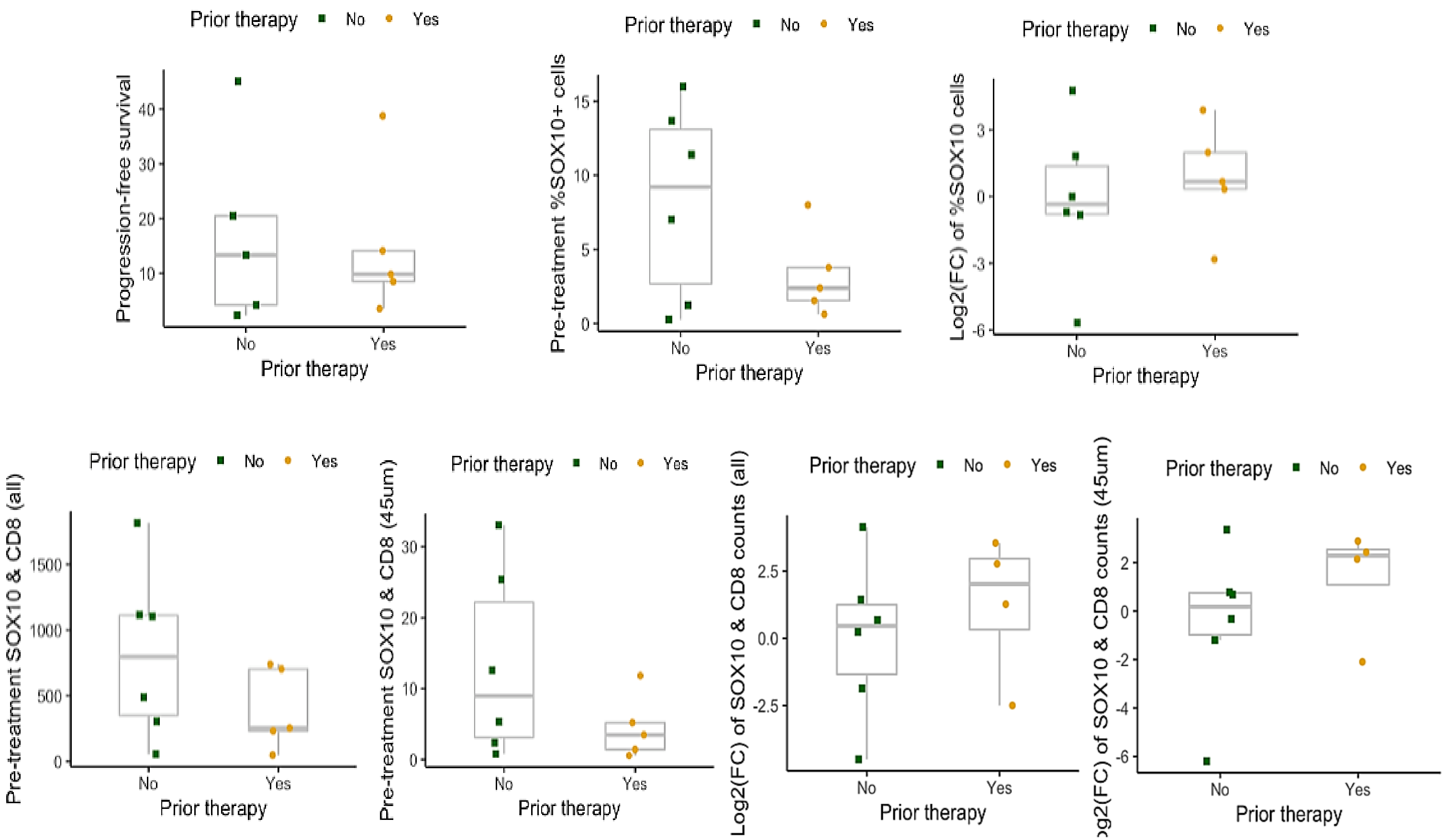

Supplementary Figure 2. Boxplots based on prior immuno- and/or chemo- therapies before BRAF/MEK inhibition. In the cohort, six patients are prior treatment naive, whereas five patients had received immunotherapy or immunotherapy-chemotherapy combination. Extensive data overlap between patients with and without prior immunotherapy or immunotherapy-chemotherapy combination were identified. Box plots represent the group median (central bar), the upper and lower quartiles (upper and lower bars), and minimum and maximum data points (whiskers with 1.5 interquartile range, IQR).

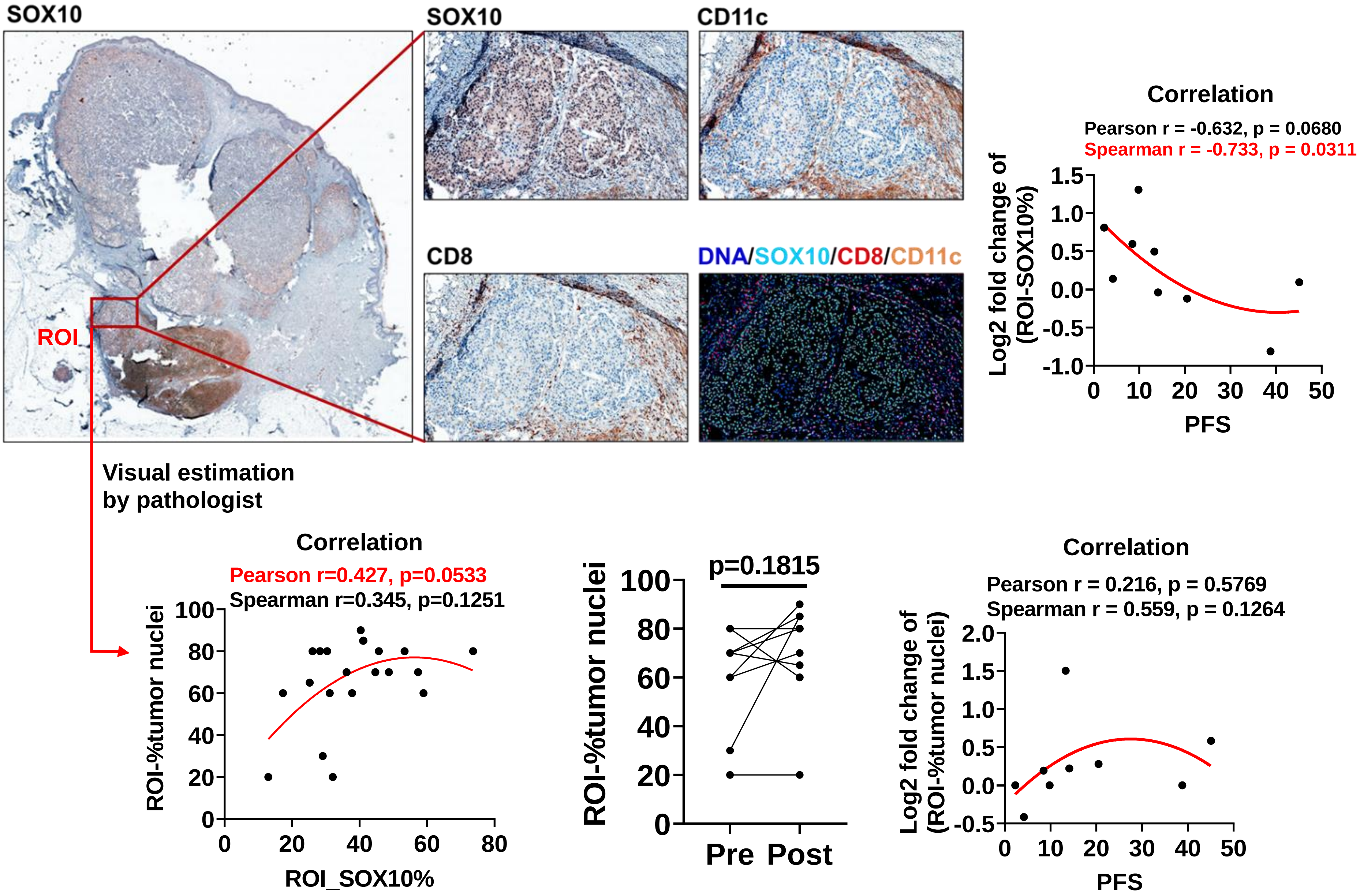

Supplementary Figure 3. No significant correlation between visual-estimated %tumor nuclei and patient's response duration to BRAF/MEK inhibition. Paired dot plot of %tumor nuclei as well as scatter plot with Pearson's and Spearman's rank correlation test were shown. Frequency (%) of $SOX10^{+}$ cells detected by immunohistochemistry staining was normalized to the count of all nucleated cells in each tumor. The values of %tumor nuclei was estimated blindly by pathologist. ROI, region of interest.

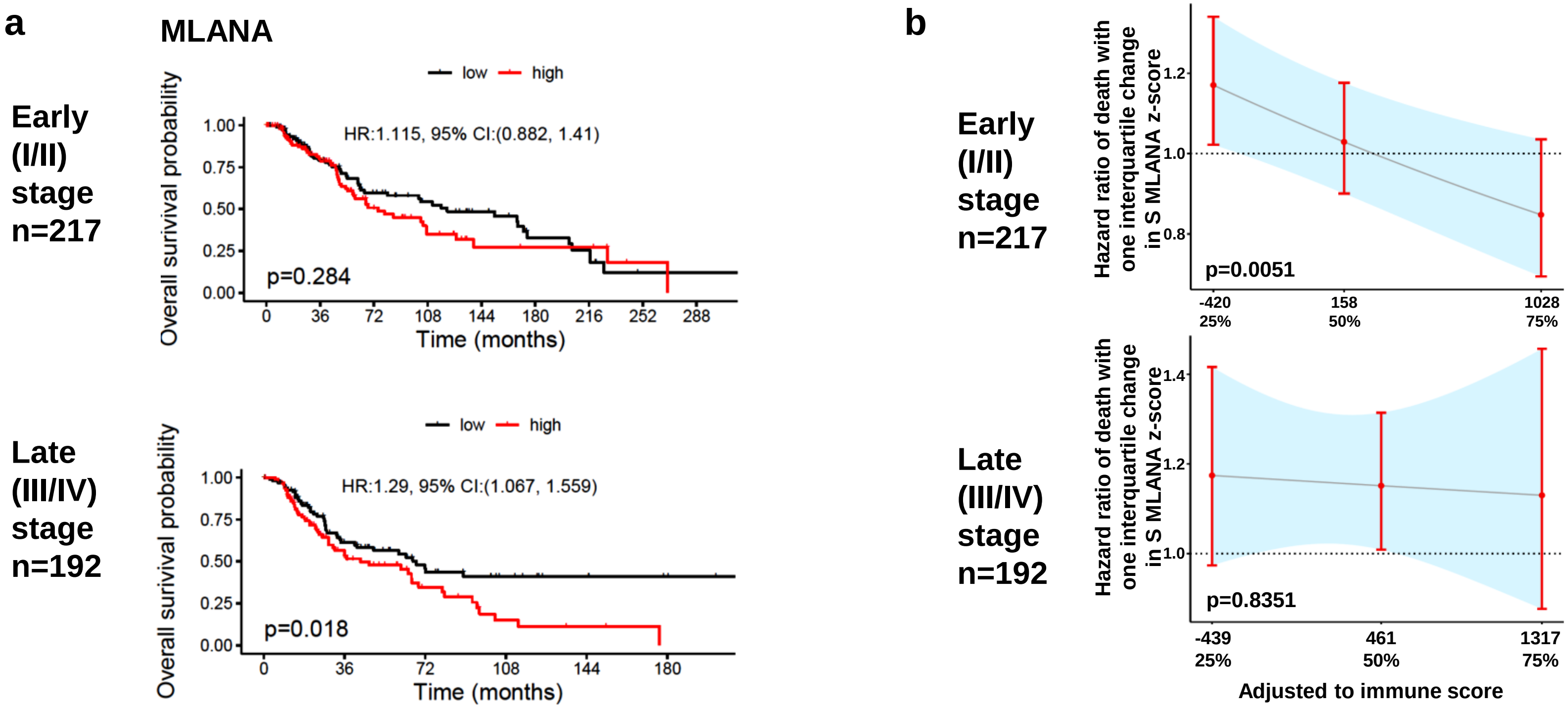

c

	Predictors in the model	C-index: Original data	C-index: Training data*	C-index: Testing data*	LR test for model comparisons
Early (I/II) stage n=217	1. Immune score	0.656	0.655 (0.596, 0.714)	0.657 (0.571, 0.735)	p=0.007
	2. Immune score + MLANA z-score	0.656	0.661 (0.600, 0.721)	0.642 (0.554, 0.731)	p=0.003
	3. Immune score * MLANA z-score (main effects and interaction effect)	0.669	0.673 (0.614, 0.728)	0.657 (0.574, 0.737)	
Late (III/IV) stage n=192	1. Immune score	0.619	0.619 (0.558, 0.683)	0.617 (0.525, 0.690)	p=0.094
	2. Immune score + MLANA z-score	0.619	0.626 (0.566, 0.685)	0.611 (0.539, 0.682)	p= 0.835
	3. Immune score * MLANA z-score (main effects and interaction effect)	0.620	0.628 (0.574 0.683)	0.606 (0.525, 0.684)	

* Adjusted C index based on internal validation with bootstrapping.

Supplementary Figure 4. Prognostic value and interaction of MLANA and immune score to predict OS in patients with melanoma. a) Survival (Kaplan-Meier) plots by predictors. b) Hazard ratio of death adjusted to immune score based on multivariable cox regression model. c) C-index by models and likelihood ratio (LR) test.

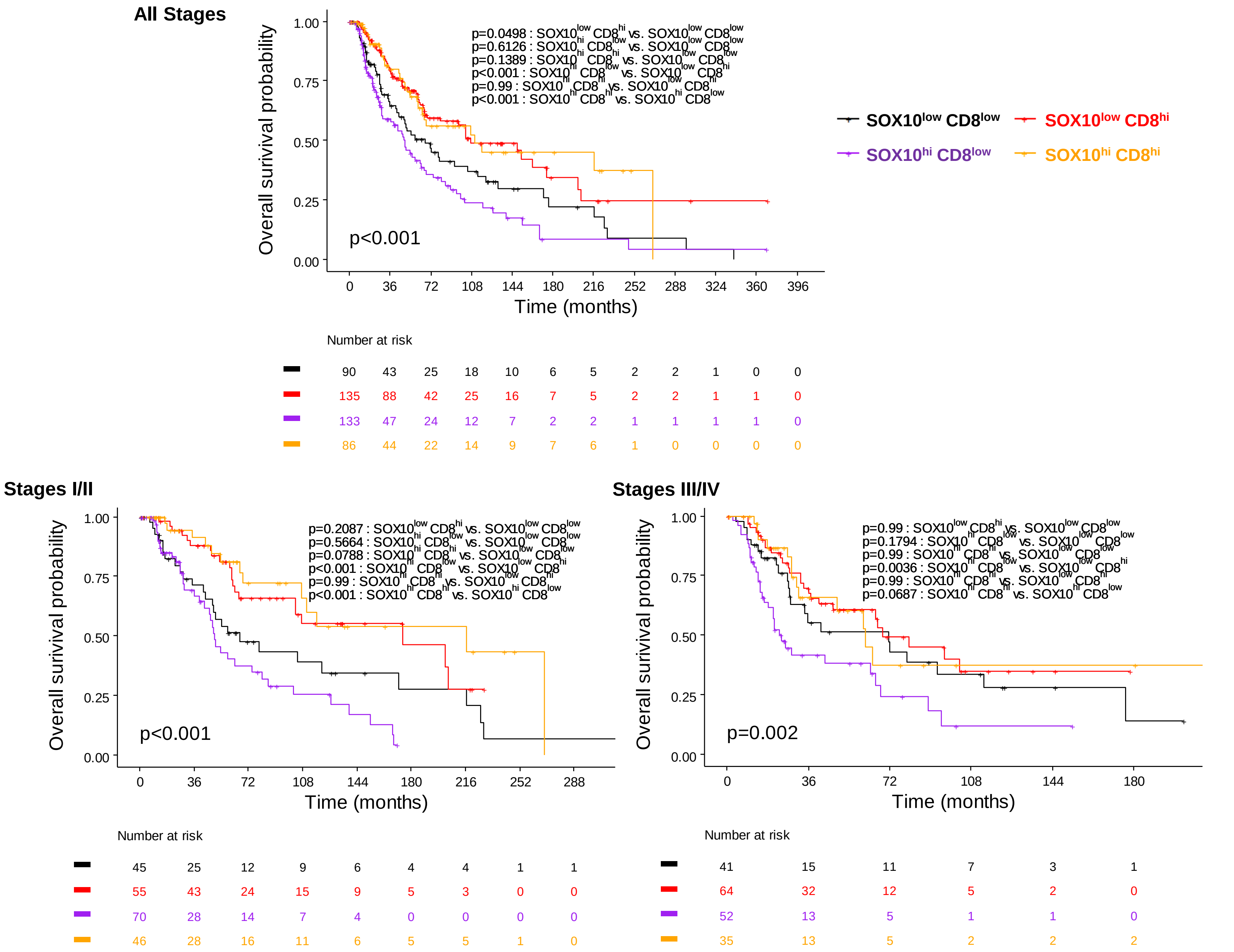

Supplementary Figure 5. $SOX10^{hi}CD8^{low}$ tumors were associated with a significant worse OS compared to $SOX10^{low}CD8^{hi}$ tumors in TCGA-melanoma patients. Survival curves were estimated using the Kaplan-Meier method and compared between groups using the log-rank test (n=445).

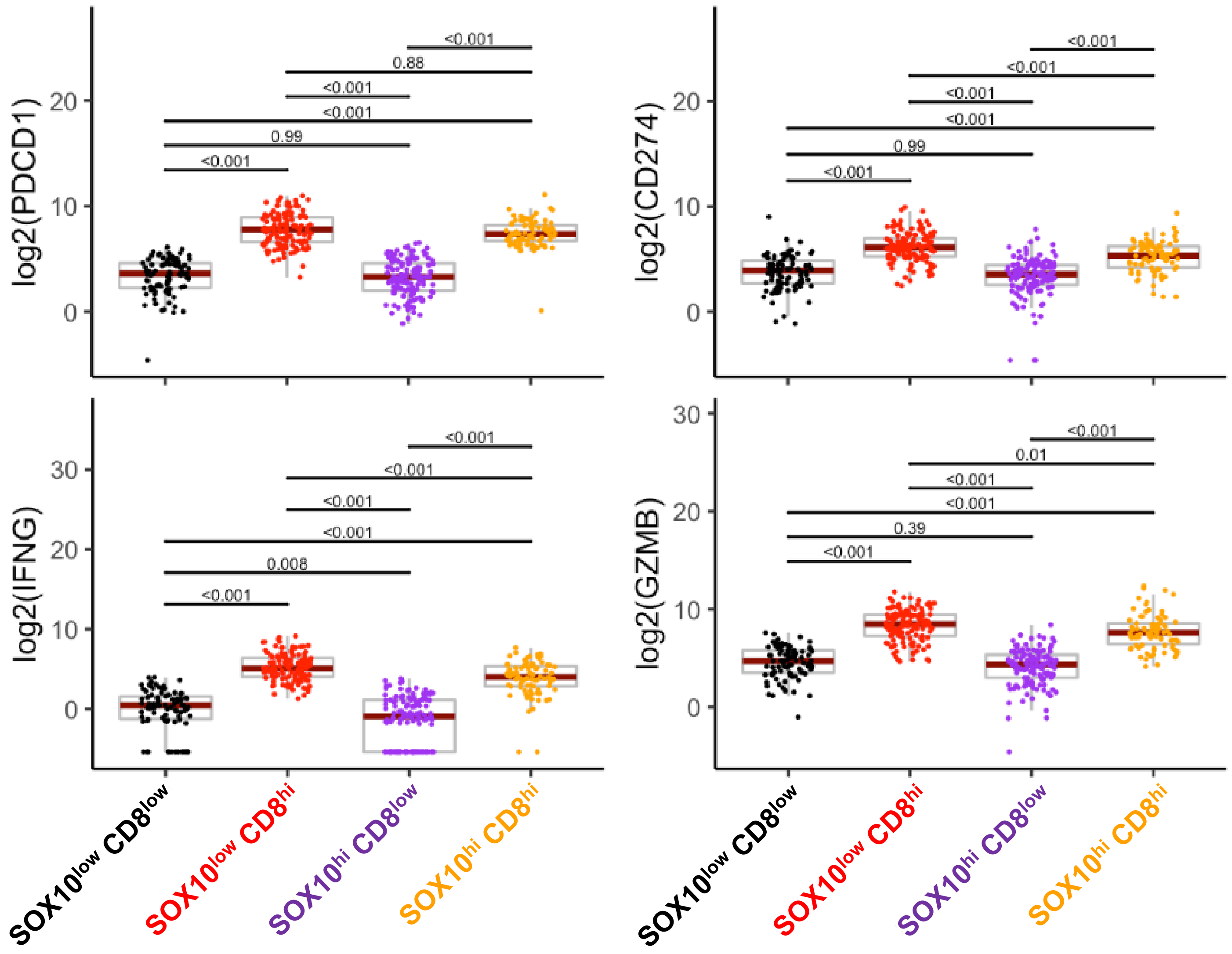

Supplementary Figure 6. Gene expression profile in tumors of TCGA-melanoma patients. Wilconxon rank sum test was used to test the difference in gene expression between groups (SOX10lowCD8low, SOX10lowCD8hi, SOX10hiCD8low and SOX10hiCD8hi). P value was adjusted with Bonferroni correction for pairwise comparisons (n=444).

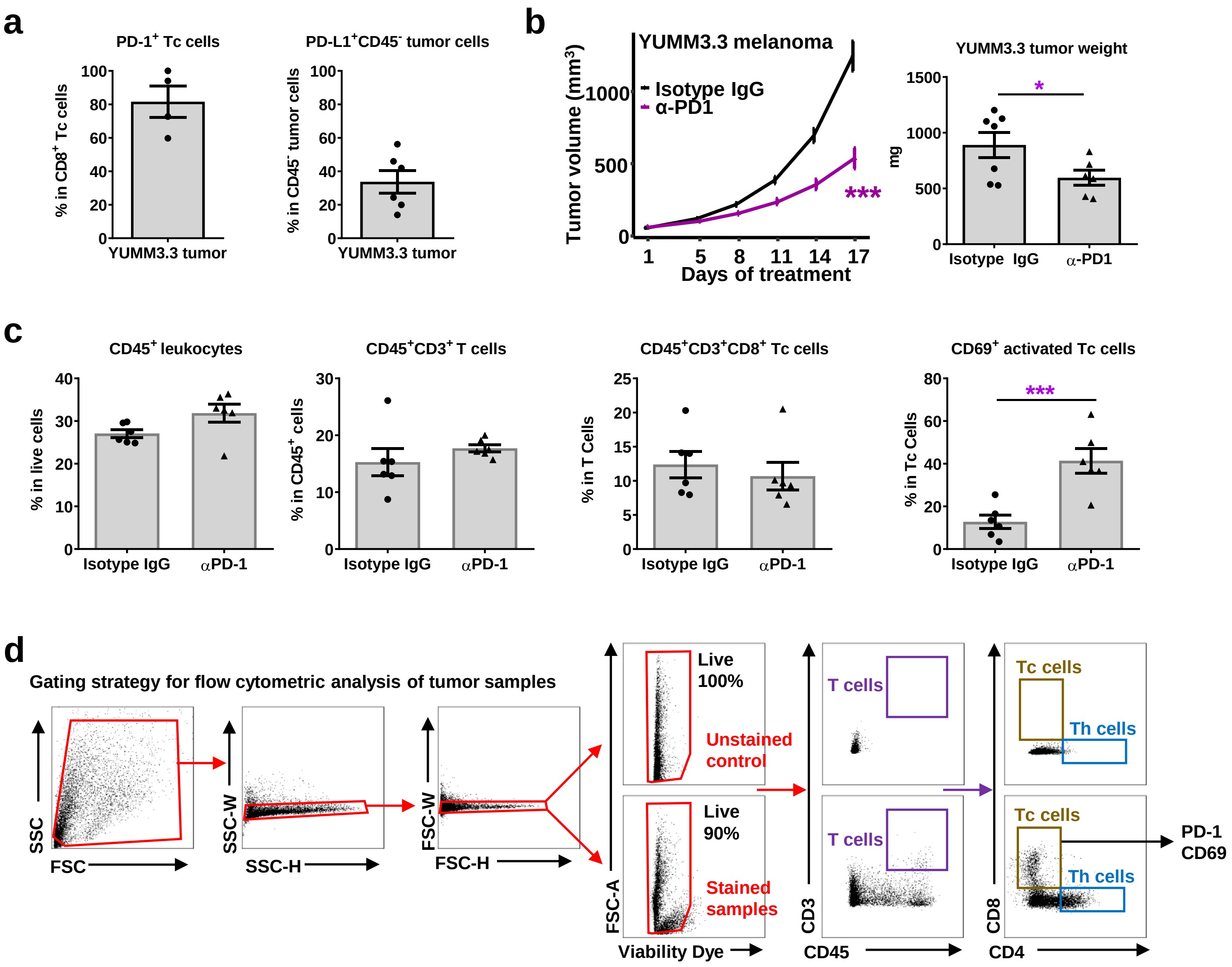

Supplementary Figure 7. PD-1 blockade inhibited YUMM3.3 melanoma tumor growth and promoted the activation of CD8+ cytotoxic T cells *in vivo.* Female C57BL/6 mice received 3×10^5 of YUMM3.3 cells in the lower back. Treatment began when tumors reached ~100mm^3 volume on average and continued until tumors in the experiment exceeded 15mm in diameter or became perforated. Anti-mouse PD-1 (clone:RMP1–14) immunotherapy or equivalent amounts of isotype control Rat IgG2a (clone: 2A3), were administered intraperitoneally at 100µg per mouse every 3 days for two weeks. a-d) Tumor samples were collected from each treatment group at day 17 of treatment and immune profiled by flow cytometry analysis. (All graphs show mean±SEM, n=6 per group.) Where indicated, * $p < 0.05$; *** $p < 0.001$. Tc, cytotoxic T. Th, T helper cells.

Supplementary Table 1. Patient characteristics and response to BRAF-targeted therapy

Median Age at Treatment Initiation, years (range)	47 (21-77)
Sex, n (%)	
Male	5 (45.5)
Female	6 (54.5)
BRAF mutation, n (%)	
V600E	10 (90.9)
V600K	1 (9.1)
Prior Treatment, n (%)	
None	6 (54.5)
Immunotherapy	4 (36.4)
Immunotherapy, chemotherapy	1 (9.1)
Targeted Therapy, n (%)	
BRAFi monotherapy	4 (36.4)
BRAKi/MEKi	6 (54.5)
BRAFi/PI3Ki	1 (9.1)
Median PFS on BRAF pathway inhibition, months (range)	14.1 (2.3-60.5)

Note: PFS (progression free survival), months. Calculated for each line of treatment with targeted therapy based on RECIST criteria and clinician review.

Supplementary Table 2. Patient characteristics of melanoma patients in TCGA-melanoma dataset

Characteristic	N	All (n = 445)[1]
**Immune score**	445	347.1 -429.6 1,236.0 (499.1 ± 1,136.3)
**Low**		223 (50%)
**High**		222 (50%)
**SOX10 z-score**	444	-0.2 -0.5 0.3 (0.0 ± 1.0)
**MLANA z-score**	444	-0.2 -0.7 0.4 (0.0 ± 1.0)
**Stage**	409	
**Early (I/II)**		217 (53%)
**Late (III/IV)**		192 (47%)
**Height (cm)**	236	170.0 163.0 178.0 (170.1 ± 9.5)
**Mutation Count**	343	290.0 123.5 530.0 (525.5 ± 1,065.8)
**BRAF Mutations**	74	
**Missense**		43 (58%)
**Wild type**		31 (42%)
**NRAS Mutations**	74	
**Missense**		9 (12%)
**Wild type**		65 (88%)
**OS month**	445	35.9 15.8 72.0 (57.9 ± 61.0)
**OS status**	445	211 (47%)
**Race**	435	
**Asian**		12 (2.8%)
**White**		423 (97%)
**Sex**	445	
**Female**		170 (38%)
**Male**		275 (62%)
**Weight (kg)**	241	80.0 69.0 90.0 (81.6 ± 19.1)
**PDCD1 z-score**	444	-0.393 -0.537 0.069 (0.006 ± 1.021)
**CD274 z-score**	444	-0.235 -0.331 0.023 (-0.024 ± 0.661)
**IFNG z-score**	444	-0.364 -0.439 -0.024 (0.014 ± 1.027)
**GZMB z-score**	444	-0.369 -0.458 0.027 (0.008 ± 1.023)

[1] **Median 25% 75% (Mean ± SD); n (%)**
