# Supplementary material for: Proximity of immune and tumor cells underlies response to BRAF/MEK-targeted therapies in metastatic melanoma patients
Source: NPJ Precis Oncol. 2022 Jan 20;6:6. doi: 10.1038/s41698-021-00249-1 (PMC8776860; doi:10.1038/s41698-021-00249-1)
Supplement: Supplementary file 2 — REPORTING SUMMARY [file 41698_2021_249_MOESM2_ESM.pdf]

nature portfolio

Corresponding author(s): Ann Richmond

Last updated by author(s): Nov 9, 2021

Reporting Summary

Nature Portfolio wishes to improve the reproducibility of the work that we publish. This form provides structure for consistency and transparency in reporting. For further information on Nature Portfolio policies, see our Editorial Policies and the Editorial Policy Checklist.

Statistics

For all statistical analyses, confirm that the following items are present in the figure legend, table legend, main text, or Methods section.

n/a	Confirmed	
☐	☒	The exact sample size (n) for each experimental group/condition, given as a discrete number and unit of measurement
☐	☒	A statement on whether measurements were taken from distinct samples or whether the same sample was measured repeatedly
☐	☒	The statistical test(s) used AND whether they are one- or two-sided<br>*Only common tests should be described solely by name; describe more complex techniques in the Methods section.*
☐	☒	A description of all covariates tested
☐	☒	A description of any assumptions or corrections, such as tests of normality and adjustment for multiple comparisons
☐	☒	A full description of the statistical parameters including central tendency (e.g. means) or other basic estimates (e.g. regression coefficient) AND variation (e.g. standard deviation) or associated estimates of uncertainty (e.g. confidence intervals)
☐	☒	For null hypothesis testing, the test statistic (e.g. F, t, r) with confidence intervals, effect sizes, degrees of freedom and P value noted<br>*Give P values as exact values whenever suitable.*
☒	☐	For Bayesian analysis, information on the choice of priors and Markov chain Monte Carlo settings
☒	☐	For hierarchical and complex designs, identification of the appropriate level for tests and full reporting of outcomes
☐	☒	Estimates of effect sizes (e.g. Cohen's d, Pearson's r), indicating how they were calculated

Our web collection on statistics for biologists contains articles on many of the points above.

Software and code

Policy information about availability of computer code

Data collection	Immune scores were derived from RNA-seq data in TCGA Skin Cutaneous Melanoma based on the ESTIMATE algorithm which were previously generated from 11 different tumor types (not including melanoma) via an R library of estimate package that is available on https://bioinformatics.mdanderson.org/estimate/rpackage.html. Clinical data (n=471) were obtained from cBioPortal (http://www.cbioportal.org/study/ summary?id=skcm_tcga). RNA sequencing-based gene expression profiling in TCGA Skin Cutaneous Melanoma were downloaded from NIH GDC website (https://portal.gdc.cancer.gov/) for survival and the immune score analysis.
Data analysis	R version 4.0.4 was used for statistical analysis.

For manuscripts utilizing custom algorithms or software that are central to the research but not yet described in published literature, software must be made available to editors and reviewers. We strongly encourage code deposition in a community repository (e.g. GitHub). See the Nature Portfolio guidelines for submitting code & software for further information.

Data

Policy information about availability of data

All manuscripts must include a data availability statement. This statement should provide the following information, where applicable:

- Accession codes, unique identifiers, or web links for publicly available datasets
- A description of any restrictions on data availability
- For clinical datasets or third party data, please ensure that the statement adheres to our policy

The datasets analysed during the current study are available in the TCGA Skin Cutaneous Melanoma dataset from cBioPortal (http://www.cbioportal.org/study/ summary?id=skcm_tcga) and NIH GDC website (https://portal.gdc.cancer.gov/). Paired advanced melanoma samples from 11 patients (pre- and post-treatment of BRAF/MEK-targeted therapy) were collected as part of NCT01205815 clinical trial. These patients were treated with BRAF/MEK inhibitor targeted therapy in the

context of standard of care treatment for metastatic melanoma. One of the study authors (DBJ) identified samples obtained during the course of clinical care from consented patients and provided de-identified clinical data for analysis.

Field-specific reporting

Please select the one below that is the best fit for your research. If you are not sure, read the appropriate sections before making your selection.

☒ Life sciences ☐ Behavioural & social sciences ☐ Ecological, evolutionary & environmental sciences

For a reference copy of the document with all sections, see nature.com/documents/nr-reporting-summary-flat.pdf

Life sciences study design

All studies must disclose on these points even when the disclosure is negative.

Sample size	Clinical data (n=471) were obtained from cBioPortal (http://www.cbioportal.org/study/ summary?id=skcm_tcga). RNA sequencing-based gene expression profiling in TCGA Skin Cutaneous Melanoma were downloaded from NIH GDC website (https://portal.gdc.cancer.gov/) for survival and the immune score analysis.
Data exclusions	Patients with overall survival (OS) less than 0, missing overall survival, or missing OS status, or patients at the stage 0, I/II NOS are excluded. After excluding 26 patients, the total patients was 445.
Replication	n/a
Randomization	n/a
Blinding	n/a

Reporting for specific materials, systems and methods

We require information from authors about some types of materials, experimental systems and methods used in many studies. Here, indicate whether each material, system or method listed is relevant to your study. If you are not sure if a list item applies to your research, read the appropriate section before selecting a response.

Materials & experimental systems

n/a	Involved in the study	
☐	☒	Antibodies
☐	☒	Eukaryotic cell lines
☒	☐	Palaeontology and archaeology
☐	☒	Animals and other organisms
☐	☒	Human research participants
☐	☒	Clinical data
☒	☐	Dual use research of concern

Methods

n/a	Involved in the study	
☒	☐	ChIP-seq
☐	☒	Flow cytometry
☒	☐	MRI-based neuroimaging

Antibodies

Antibodies used	MxIHC slides were incubated with primary anti-human antibodies, including anti-CD8 (Cat: MM39-10, McKinney, TX), anti-SOX10 (Cat: PA0813, Cell Marque, Rocklin, CA), anti-CD40 (Cat: ab13545, Abcam, Cambridge, MA), anti-CD80 (Cat: 134120, Abcam, Cambridge, MA) or anti-CD11c (Cat: PA0554, Lecia, Buffalo Grove, IL).<br>Immunotherapy anti-mouse PD-1 (clone: RMP1-14), or equivalent amounts of isotype control Rat IgG2a (clone: 2A3), were administered intraperitoneally at 100µg per mouse every three days for two weeks. Both antibodies were purchased from BioXcell (Lebanon, NH).<br>Flow cytometric antibodies include CD45-APC/Cy7 (BioLegend, #103116, 1:250), CD3-PerCP/eFluor 710 (eBioscience, #460032-80, 1:200), CD4-BV421 (BioLegend, #100438, 1:200), CD8-AlexaFlour700 (BioLegend, #100729, 1:500), and CD69-APC (BioLegend, #104513, 1:100).
Validation	All MxIHC antibodies were validated by Translational Pathology Shared Resource (TPSR) at Vanderbilt University. Antibodies for the immunotherapy and flow cytometric analysis were previously tested and reported by the Richmond lab.

Eukaryotic cell lines

Policy information about cell lines

Cell line source(s)	YUMM3.3 cells were provided by Marcus Bosenberg (Yale University).

Authentication	The BRAF genetic mutations of the YUMM cell lines was verified by response to BRAF/MEK inhibitors and by RNAseq.
Mycoplasma contamination	Cells were tested negative for mycoplasma contamination.
Commonly misidentified lines (See ICLAC register)	n/a

Animals and other organisms

Policy information about studies involving animals; ARRIVE guidelines recommended for reporting animal research

Laboratory animals	C57BL/6 mice were purchased from Jackson Labs. Tumor xenografts were established in 7 week-old female mice.
Wild animals	The study did not involve wild animals.
Field-collected samples	The study did not involve samples collected from the field.
Ethics oversight	Animal studies were approved by the Vanderbilt Institutional Care and Animal Use Committee (IACUC) and were performed in accordance with Vanderbilt IACUC guidelines. All animals were housed under pathogen-free conditions at the Vanderbilt Animal Care Facility.

Note that full information on the approval of the study protocol must also be provided in the manuscript.

Human research participants

Policy information about studies involving human research participants

Population characteristics	Of the 11 patients, the median (range) age was 47 (21–77) and five were men (45.5%). Of our cohort, four (36.4%) were treated with BRAF inhibitors alone, four (36.4%) were treated with dual BRAF and MEK inhibition, and three (27.3%) were treated with single-agent BRAF inhibition with change to dual therapy (BRAF/MEK or BRAF/PI3K). All patients had complete or partial response, with the exception of one patient (9%) with mixed response, and the median PFS was 11.6 months for the entire cohort. Data Collection: 6/3/2010-1/1/2022.
Recruitment	Patient samples were collected as part of NCT01205815 clinical trial on a tissue-collection protocol approved by the Vanderbilt University IRB.
Ethics oversight	Vanderbilt-Ingram Cancer Center and Vanderbilt IRB.

Note that full information on the approval of the study protocol must also be provided in the manuscript.

Clinical data

Policy information about clinical studies

All manuscripts should comply with the ICMJE guidelines for publication of clinical research and a completed CONSORT checklist must be included with all submissions.

Clinical trial registration	NCT01205815
Study protocol	clinicaltrials.gov/ct2/show/study/NTC01205815
Data collection	6/3/2010-1/1/2022
Outcomes	Non-interventional study; ongoing. Data included does NOT come from a randomized clinical trial. As such, no CONSORT document should be required.

Flow Cytometry

Plots

Confirm that:

- [ ] The axis labels state the marker and fluorochrome used (e.g. CD4-FITC).
- [x] The axis scales are clearly visible. Include numbers along axes only for bottom left plot of group (a 'group' is an analysis of identical markers).
- [ ] All plots are contour plots with outliers or pseudocolor plots.
- [x] A numerical value for number of cells or percentage (with statistics) is provided.

Methodology

Sample preparation	Isolated cells were incubated with Ghost Dye Violet 510 (Tonbo Biosciences) to discriminate live/dead cells and washed with PBS containing 1% v/v FBS. After blocking Fc receptors with anti-mouse CD16/CD32 mAb (BD Biosciences) for 20 minutes,

cells were incubated with target antibodies, including CD45-APC/Cy7, CD3- PerCP/Cy5.5, CD4-BV421, CD8-AlexaFlour700, and CD69-APC. All flow cytometric antibodies were purchased from BioLegend (San Diego, CA). After staining, cells were washed twice in PBS containing 1% v/v FBS and fixed with 1% formalin in PBS.

Instrument

BD LSR Fortessa flow cytometer

Software

FlowJo software (Version 10.5.3)

Cell population abundance

Using YUMM3.3 (BRAFmut) melanoma model in C57BL/6 mice, we found that ~30% of CD45- cells, including melanoma cells, are PD-L1+, while ~80% of CD8+ T cells are PD-1+ in the tumor microenvironment (TME) (Suppl.Fig.7A). Two weeks of anti-PD1 treatment resulted in a significant (>50%) inhibition of tumor growth (Suppl.Fig.7B). Anti-PD1 treatment does not alter the frequency of total CD45+ leukocytes, total CD3+ T cells, or CD8+ T cells in the TME (Suppl.Fig.7C). However, while ~10% of CD8+ T cells are activated (CD69+) in the tumors treated with isotype control IgG, anti-PD1 treatment increased the frequency of activated CD8+ T cells to ~40% in the TME (Suppl.Fig.7C).

Gating strategy

Detailed gating strategy was shown in Supplementary Figure 7D.

☒ Tick this box to confirm that a figure exemplifying the gating strategy is provided in the Supplementary Information.
